# Mental Health First Aid crisis role-plays between pharmacists and simulated patients with lived experience: a thematic analysis of debrief

**DOI:** 10.1007/s00127-023-02443-x

**Published:** 2023-03-16

**Authors:** Ricki Ng, Claire L. O’Reilly, Jack C. Collins, Helena Roennfeldt, Sara S. McMillan, Amanda J. Wheeler, Sarira El-Den

**Affiliations:** 1 Faculty of Medicine and Health, Pharmacy and Bank Building A15, The University of Sydney School of Pharmacy, The University of Sydney, Science Road, Camperdown, NSW 2006 Australia; 2 Menzies Health Institute Queensland, Nathan Campus, Griffith University, Nathan, Australia; 3 School of Pharmacy and Medical Sciences, Griffith Health, Griffith University, Gold Coast, Australia; 4 Faculty of Medical and Health Sciences, University of Auckland, Auckland, New Zealand; 5 Centre for Mental Health, Griffith University, Brisbane, Australia

**Keywords:** Mental health, First aid, Pharmacist, Crisis intervention, Suicide, Simulation training

## Abstract

**Purpose:**

Healthcare professionals, including pharmacists, can recognise and assist people experiencing mental health crises. Despite this, little is known about how pharmacists assist and engage with people presenting with signs and symptoms of mental health crises. This study aimed to (i) examine pharmacists’ mental health crisis assessment language during simulated patient role-plays (SPRPs) and (ii) explore participants’ experiences of participating in SPRPs of Mental Health First Aid (MHFA) scenarios.

**Methods:**

Fifty-nine MHFA-trained pharmacy staff participated in audio-recorded SPRPs of three crisis scenarios enacted by a mental health consumer educator (MHCE). Post-SPRP, pharmacy staff members (including role-playing and observing participants), engaged in reflective debrief discussions with the facilitator and MHCEs. Debrief discussions were transcribed verbatim and analysed using inductive thematic analysis and suicide assessment language was explored.

**Results:**

The majority of role-playing pharmacists asked about suicidal ideation using appropriate, direct language (*n* = 8). Qualitative analyses of debrief discussions yielded four themes: (i) Relationship with the consumer, (ii) Verbal and non-verbal communication, (iii) Challenges with crisis assessment, which included difficulties associated with initiating conversations about suicide and mania, and (iv) Reflective learning.

**Conclusion:**

While pharmacists demonstrated the appropriate suicide assessment language post-MHFA training, pharmacists felt uncomfortable initiating conversations around suicide and lacked confidence during crisis assessments. SPRPs provided pharmacists with opportunities to reflect on and practice MHFA skills in a safe learning environment. Future research exploring how MHFA training and SPRPs impact pharmacists’ ability to provide MHFA in real-world settings is warranted.

## Introduction

A mental health crisis, such as suicidal thoughts and behaviours, panic attacks or severe psychotic states [1], is defined as a “situation in which a person’s behaviour puts them at risk of hurting themselves or others and/or prevents them from being able to care for themselves or function effectively” [2]. Suicide is a significant public health concern globally, contributing to more than 700,000 deaths each year [3]. It is also one of the leading causes of death globally, with a 30% increase in the suicide rate over the past 20 years [4].

Preventing suicide requires an active, multifaceted approach whereby suicide assessment and early intervention are integrated within healthcare services [5]. Healthcare professionals’ lack of training in suicide care can result in an inability to respond to warning signs and stigma towards people experiencing mental illness, which can delay or prevent help-seeking and recovery [6–8]. Healthcare professionals, including pharmacists given their frontline primary healthcare roles, are in a unique position to support people in distress and recognise changes in medication use, mood or behaviour. Pharmacists can also support people in distress and connect consumers to the services they need [9, 10]. To demonstrate the importance of early intervention in primary care, evidence suggests that on average 80% of people contacted a primary healthcare provider in the year prior to suicide, and 44% in the month before suicide [11]. Similarly, research among pharmacists indicates that 85% of pharmacists have interacted at least once with someone at risk of suicide; hence, training pharmacists to assess for suicidal intent and intervene appropriately enables them to contribute to suicide prevention [10, 12].

Training in suicide assessment and intervention even among mental healthcare professionals is currently not sufficient [13]. Similarly, pharmacists and pharmacy staff members have reported feeling unprepared and ill-equipped when describing their experiences identifying, caring for and supporting people at risk of suicide [14, 15]. One way to ensure pharmacists have appropriate skills to confidently intervene is through Mental Health First Aid (MHFA) training. MHFA is an accredited training program designed to teach participants how to assess and assist those experiencing mental health problems (e.g. symptoms of depression, anxiety, substance use disorders) and crises [1]. In some settings, MHFA training has been embedded into tertiary curricula [16, 17], and evaluations demonstrate that it can lead to improvements in attitudes, knowledge and confidence among students [18]. However, self-reported confidence does not always translate into improved behaviours and MHFA participants require further opportunities to practice their newly acquired skills in safe learning environments [19].

Simulation-based education is an effective teaching strategy that allows learners to participate in realistic clinical scenarios that reflect real-world situations. It has been used in a range of healthcare settings, including medicine [20], nursing [21] and pharmacy [22, 23] to improve communication skills and behaviour. Effective communication in crisis assessment, particularly regarding the importance of asking directly about suicidality has been well documented [1]. However, Murphy et al. demonstrated that some pharmacists still feared to ask questions about suicide directly, despite completing training [7]. This further demonstrates the need for not only training and education, in this area, but also opportunities to practice these skills. Several studies have shown the benefits of simulation among pharmacy students in improving their confidence to assist with mental health problems and crises [19, 24]. However, to our knowledge, there has been no published research that explores pharmacists’ ability to demonstrate their MHFA skills post-training, such as through simulated patient role-plays (SPRPs). Therefore, the purpose of this study was to explore pharmacists’ mental health crisis assessment language during SPRPs, and participants’ experiences of participating in SPRPs of MHFA crisis scenarios.

## Methods

### Participants

Pharmacy staff who participated in the SPRP sessions were recruited as part of the *PharMIbridge* Randomised Controlled Trial (RCT) and this process is described in detail elsewhere [25]. Participants of the SPRP sessions were staff of community pharmacies randomised to the Intervention Group including pharmacists, pharmacy interns, assistants, or technicians. Ethical approval was granted by the Griffith University Human Research Ethics Committee (Project number: 2019/493).

### Procedures

Five *PharMIbridge* training sessions were held across four Australian regions in three states and territories, from September 2020 to February 2021. Each training session included one 90-min SPRP session which involved role-playing three scenarios followed by a debrief discussion. Participants attended MHFA training immediately before the SPRP session, whereby attendance was required for participants who did not have existing MHFA accreditation, as part of their participation in the *PharMIbridge* RCT.

A facilitator (registered pharmacist and MHFA instructor) attended all sessions. Five different people with a personal lived experience of mental illness or caring for others with lived experience of mental illness (hitherto referred to as mental health consumer educator or MHCE) were involved in the SPRP and debrief discussion. MHCEs were briefed, provided with the scenario and an opportunity to ask questions and practice the scenario, prior to each session. MHCEs participated as the simulated patient for all three scenarios within the session they attended.

#### Scenarios and marking rubric

The three scenarios used in the SPRP sessions were adapted from previously developed scenarios, used to assess pharmacy students’ MHFA skills through observed behavioural assessments [19, 26]. Two scenarios were in relation to suicide; one simulated patient admitted to experiencing suicidal thoughts with a plan and the second had warning signs of suicide but had no suicidal thoughts nor a plan. The third scenario involved an acute episode of antidepressant-triggered mania, whereby the simulated patient exhibited signs of mania such as having increased energy and euphoria.

For each scenario, a purpose-designed assessment rubric was used to assess the pharmacists’ performance during the SPRP. These assessment rubrics were developed based on the MHFA Action Plan and a scoring system developed by MHFA researchers [19]. The marking rubrics have been psychometrically tested for validity and reliability and have been used to assess pharmacy students’ performance in SPRPs [18, 19, 26]. In the current study, the marking rubrics were used by the role-playing pharmacist for self-assessment post-SPRP, as well as by the facilitator and MHCE to assess the role-playing pharmacist who participated in the SPRP (hitherto referred to as the ‘RP’). In line with the study’s aim of exploring pharmacists’ experiences of participating in SPRPs, scores were not analysed quantitively, and the scores and rubrics were used only as a reflective tool to facilitate the debrief discussions.

### Simulated patient role-play sessions

During each SPRP and debrief session, participants either volunteered to participate in the role play or observe the simulated scenario. At the end of each SPRP, the RP, MHCE and facilitator were each given a scenario-specific marking rubric to complete and assess each RP’s performance during the SPRP. The remaining participants (pharmacists or pharmacy staff members) who observed the simulation (hitherto referred to as the observing participants (‘OPs’)) were given the opportunity to participate and provide feedback to the RP during the group debrief discussion.

### Group debrief discussions

Upon completion of each SPRP, debrief discussions were initiated by the facilitator. Each debrief discussion involved active dialog among the RPs and OPs, as well as the MHCE and the facilitator, who functioned as content expert to advance discussion when needed. Group debrief discussions were audio-recorded with participant consent.

### Data analysis

Audio-recorded debrief discussions were de-identified and transcribed verbatim. To assess the language used by the RPs during the mental health crisis assessments, the terminology used to assess suicide was classified as either ‘direct’ or ‘indirect’ suicidal language according to MHFA principles as well as previous research exploring suicide language [1, 19]. The language was considered to be ‘direct’ if the participant inquired about suicidal thoughts and behaviours directly using terms such as “suicide” or “killing yourself” which are non-ambiguous in terms of their intention to enquire about end-of-life [1]. Participants’ language was classified as ‘indirect’ if ambiguous terms such as “harming yourself” or “dark thoughts” were used.

Thematic analysis of debrief discussions was conducted to identify patterns of meaning across the data. Due to the lack of prior research in the area of debrief analysis, an inductive thematic analysis approach was used whereby findings were driven by the data [27, 28]. Analyses were informed by the step-by-step guidance for conducting data analysis, outlined by Braun and Clarke [28]. First, one member of the research team (RN, a pharmacist and MHFA instructor) read and re-read the transcripts to become familiar with the data. Personal reflexivity was taken into consideration throughout the process of data analysis. Second, five transcripts were coded by RN iteratively and then discussed in meetings with two other registered pharmacists who are also mental health researchers (SE, a Master MHFA instructor, and JC who is MHFA-trained). Through these discussions, a more extensive coding framework was developed that was used to code the entire data set, with additional codes generated as necessary, in collaboration with three researchers (SE, JC and COR, who is also a Master MHFA instructor). Codes were then organised into preliminary themes through an iterative process amongst the authors (RN, SE, JC and COR), and further themes were developed. The research team met regularly to review and discuss the codes and themes and the final themes were developed. Verbatim quotes were extracted to exemplify the themes. The research and reporting process of the debrief analysis was guided by the Standards for Reporting Qualitative Research (supplementary material DS1) [29].

## Results

Fifty-nine pharmacy staff, including 55 pharmacists and four pharmacy assistants or technicians attended one of the five SPRP sessions. The average length of the sessions was 78.2 min. Participants were primarily female (61.0%), with an average age of 36.7 years (SD = 10.8). The majority were born in Australia (71.2%). More than half of the participants (52.5%) worked for five years or less in their current role in community pharmacy. Less than half of the participants (44.1%) completed MHFA training previously. Nineteen participants (32.2%) reported having personal lived experience with mental illness.

### Mental health crisis (suicidal) assessment in simulated patient role-plays

Of the 10 RPs that conducted suicidal crisis assessment, eight RPs demonstrated the use of direct suicide-specific terminology, while two used indirect, ambiguous terms. Table 1 shows examples of pharmacists’ suicide assessment language used during the SPRPs.

**Table 1:** Examples of direct and indirect suicide assessment language used during SPRPs

Direct	Indirect
*“Have you thought about…suicide? And…do you have a plan?”* (RP1.2)	*“Have you been thinking of harming yourself?… Any dark thoughts along those lines?”* (RP2.2)
*“When someone's feeling so flat and numb, sometimes people can have thoughts of killing themselves because they feel like that's the only way out. You ever had any suicidal thoughts or anything like that?”* (RP3.4)	*“You mentioned a lot of ideas. Have you had any thoughts you know that impact on your safety?”* (RP3.4)

### Group debrief discussion analysis

Four themes were generated by the inductive thematic analysis. Three overarching interrelated themes emerged from the data, in that the *(i) Relationship with the consumer* was seen as a facilitator, which, if supported by *(ii) Verbal and non-verbal communication*, could help with the *(iii) Challenges with crisis assessment*. Challenges included using appropriate language to assess for suicide, recognising the risk of crises and knowing when to refer to other healthcare professionals, if needed. A fourth theme relating to how the SPRPs encouraged *(iv) Reflective learning* also emerged.

#### Relationship with the consumer

Participants reported that good professional relationships and rapport with consumers were beneficial as they both enabled consumers with mental illness to feel comfortable in approaching the pharmacist and allowed the pharmacist to identify changes in behaviour or signs of worsening mental health in consumers. Pharmacists believed that the rapport that they build with their consumers through casual conversations allows them to pick up any signs that may indicate a possible mental health crisis:

*“It is generally more natural with a customer that you've known…because you're comfortable talking to them. And you generally know the personality…I think it's quite hard if you don't know the person.”* (RP1.2).

Pharmacists emphasised that trust was a prerequisite to initiating conversations related to mental health crises. Pharmacists felt insecure about how the consumer was feeling, especially if the consumer was not well-known to the pharmacy, and acknowledged that communication would be superficial if pharmacists did not manage to establish a two-way conversation:

*“I think the start* [of the interaction during the SPRP] *was a bit awkward. I think it's always hard…because you don't ever expect it at work…it’s hard to try and get the ball rolling when it's someone you don't know.”* (RP1.3).

MHCEs also gave feedback to participants about how to establish a professional relationship and trust with consumers:

*“In terms of building rapport my idea is that you* [pharmacy staff] *offer enough that’s professional but is also human. So, there’s your professional role. You do that, but you also do some human things that show that you care.”* (MHCE1).

MHCEs emphasised the need to provide continued support by not only providing realistic solutions to consumers but also supporting them by listening attentively and expressing a genuine interest in their struggles:

*“I just felt part of empathy is just listening…empathy has a lot to do with listening.”* (MHCE4).

#### Verbal and non-verbal communication

The importance of communication methods was discussed among RPs and MHCEs. MHCEs recognised the importance of using conversational language when asking about mental health problems and crises:

"*Just weave it into normal conversation that doesn't sound like a line of questioning”* (MHCE2).

On the other hand, RPs were conscious of using the appropriate language relating to suicide:

*“I tried my best to not use language that was stigmatising. So, I didn't say the words commit suicide. I just said considered suicide…which is something that I've actually had to get out of a habit of saying.”* (RP1.1).

While it was encouraging that RPs recognised that some words may have stigmatising connotations, it is important to note that they still struggled when communicating during the SPRP. As described by a RP, it was:

*“Quite hard to use the right words without using the wrong words”*. (RP1.5).

In addition to verbal communication considerations, MHCEs emphasised the importance of non-verbal communication. Interpersonal elements such as *“just sit[ting] quietly”* (MHCE1) and *“[having] eye contact”* (MHCE4) were thought to help build rapport and increase consumer trust in crisis risk assessment. MHCEs noted the importance of being calm and allowing for pauses:

*“Simplify your language, keep your body and your voice calm and really think about when you are going to speak and how long the gaps are. Make sure you leave some silence and just be supportive.”* (MHCE1).

RPs also recognised that their body language impacted how they might be perceived by consumers. Feelings of concern over pharmacists’ facial expression and poor posture were raised, and pharmacists sought advice from MHCEs about these issues:

*“Can I just ask you* [MHCE]*…often with me, you can see my cogs quickly. And I know that and I'm just wondering for people with mental health issues do they find that a bit skipped? Is that a fair enough thing to do? Or is that terrifying?”* (RP2.2).

#### Challenges with crisis assessment

Encountering mental health crisis situations in a pharmacy was viewed as a challenging experience for pharmacy staff. Many expressed uncertainties about how to initiate and conduct useful conversations that would not cause discomfort or further distress among consumers. Both RPs and OPs agreed that it was often hard to determine when to actively assess for suicide risk and acknowledged that suicide is an emotionally loaded subject:

*“I find it quite hard to raise that suicidal issue…sometimes I find that hard to bring that question into the conversations.”* (OP5.3).

Pharmacists considered whether the seriousness of suicide made it difficult for consumers to be honest about their feelings and whether consumers may present at the pharmacy in a manner that may not initially alert the pharmacist:

*“In those more mild situations just in pharmacy, it’s hard to delve straight into the serious questions around ‘Have you thought of suicide?’ Because it might seem completely out of kilter.”* (RP3.5).

Participants felt that differences in presentation among people at risk of suicide also contributed to the challenge of identifying individuals who may be at risk. When reflecting on past experiences of encountering consumers who have attempted suicide, a RP expressed that some consumers may not show distinctive signals indicating suicidality, which revealed the complexity around suicide:

*“We’ve got two patients that have committed suicide…One showed absolutely no symptoms, at all. You have a chat with him every week, talking about their family. And then within days, police came, and he had actually [died by suicide] … But did not present warning signs. Nothing that you picked up, no struggles. We don't believe even to the day, I don't even know what snapped. He just died. And another patient…he actually did come in crying”* (RP2.3).

Furthermore, despite completing MHFA training, there were concerns about potential harm resulting from discussing suicidality:

*“I didn't want to* [ask about suicide] *because I didn't want her to think about it. Because some people don't actually think about that until you put it in front of them. And then you kind of put an idea in their head.”* (RP2.3).

Nonetheless, participants agreed on the importance of initiating a conversation about suicide with people at risk and the need to ask about suicide directly. However, the level of confidence when conducting suicide assessments varied among participants, with some indicating that these assessments were conducted *“fairly regularly in the pharmacy”* (RP1.4). On the other hand, some OPs questioned whether it would be appropriate to ease into the conversation by conducting indirect questioning first and then using direct language to assess for crisis:

*“Would you tiptoe around the edges to see what the response is and then ask the direct question? Would you guys feel comfortable in asking the indirect questions, see what the response is …and then ask a direct question…based on the response to that. Is that … okay to do? Because not every time do you think that someone's going to walk out of here and pop a 100 Panamax* [to attempt suicide]*?”* (OP7.3).

Uncertainty about consumers’ reactions to receiving mental health support was another challenge. For example, pharmacists worried that they would *“dampen her mood”* and *“did not feel that comfortable with trying to bring her back to reality”* (RP3.2) when assessing for crisis during the SPRP of the mania scenario. RPs questioned how consumers might respond to their offer to connect them with appropriate professional help when experiencing a crisis:

*“When you are so just ridiculously confident that…your mind is clear…how receptive is someone like that to…a helpline?”* (RP3.2).

Participants also acknowledged the difficulty in providing realistic professional help options and relaying these to the simulated patient, especially in relation to the mania scenario:

*“It's always hard to bring people down, apart from sending* [them] *to hospital for a short stay and come back under control. It's not the first thing you can say to them.”* (RP3.3).

#### Reflective learning

Pharmacists expressed satisfaction with their educational experience during the SPRP sessions and discussed how similar the scenarios were to their real-life practice in community pharmacy. Although RPs found *“the scenario* [to be] *difficult with the audience”* (RP1.2), the SPRP experience involved “*a good scenario, good patient”* whereby some pharmacists thought* “that's what we'll be faced with”* (RP1.5). The overall quality and authenticity of the scenarios meant that they reflected real-life presentations:

*“I think this scenario was something that we've seen versions of in the pharmacy before.”* (RP3.5).

Upon reflecting on their performance using the rubric, RPs reflected that their *“history taking wasn’t great”* and could have improved on their *“*[information gathering in] *a more systematic way”* (RP2.1). SPRPs provided RPs with a unique opportunity to consider their strengths, weaknesses and learning needs:

*“I definitely reading* [the rubric] *I’ve got some improvements to make. I think that I could have done better that like how can I help and* [use] *more open ended questions…I could focus more on how she was feeling rather than what do we do next.”* (RP1.1).

Similarly, RPs reflected on how to identify possible crisis signs earlier.

*“I don't think I was quick enough in understanding to start with. I was asking like how you* [MHCE] *are going and you kept on repeating I've already told you that…. So I probably didn't pick up on cues, quick enough”* (RP1.2).

One RP reflected on the difficulty of explaining the crisis situation and their concerns during the SPRP to someone experiencing symptoms of mania:

*“I didn't identify the problem for a while…I was just like you're feeling great, I'm feeling great…*[I] *feel like she was in a bit of a state of mania, then it was kind of a mixed bag. She didn't seemed flat or upset. So like with people* [being] *happy and a bit excited, it's really hard to judge that they've got a problem.”* (RP3.3).

## Discussion

The majority of pharmacist participants assessed for suicide using direct, appropriate suicide terminology. The four themes generated from the thematic analysis of the debrief discussions provide unique insights into the experiences and reflections of pharmacy staff through simulations of mental health crises. This study demonstrates that MHFA-trained pharmacy staff recognise the need to establish rapport with mental health consumers, the importance of verbal and non-verbal communication, and the challenges associated with assessing for mental health crises. The feelings of uncertainty around managing crises discussed during the debrief discussions illustrate the need for further opportunities to practice and reflect on MHFA skills in safe learning environments. Furthermore, RPs’ responses during the debrief discussions highlighted that the SPRPs were reflective of their real-world experience of working in community pharmacy and that the authenticity of the session allowed them to engage in active learning while reflecting on their current and future practice.

The involvement of MHCEs provided pharmacy staff members an opportunity to interact with people with lived experience and to have a first-hand understanding and appreciation of the perspective of people with lived experience of mental illness and their carers. As seen in the study, the interaction that pharmacy staff members had with MHCEs allowed pharmacy participants to ask questions to address the associated challenges during crisis assessments. Previous evidence also supports the importance of involving MHCEs in education as these interactions may help reduce mental health stigma and improve empathy and attitudes among participants [30], and such benefits are not replicated in traditional didactic forms of education [24]. While not assessed in this study, the inclusion of MHCEs may provide personal benefits towards MHCEs themselves. Previous studies exploring MHCEs involvement in SPRPs showed that their involvement in mental health education can positively impact their sense of self-esteem and empowerment as well as potentially support their future help-seeking and recovery journey [30].

The World Health Organisation recognises the importance of communication skills among mental healthcare professionals to ensure high-quality mental healthcare [31]. RPs were able to identify strengths and weaknesses in their communication skills by actively participating in the SPRPs. These findings are consistent with previous studies, which have identified that participants who take part in simulations value the opportunity to practice communication skills, engage in reflective learning and transfer what was learned to practice [32]. Self-reflection fostered a greater appreciation of the need for better verbal and nonverbal communication skills and the importance of listening and adopting a consumer-centered approach. Similarly, other studies have shown that pharmacy students acquired a range of skills, including communication skills and being able to deal with difficult situations, after participating in simulation [33, 34].

The results of the current study also highlight the importance of building a good therapeutic relationship with consumers, which is in line with previous studies among healthcare professionals highlighting the importance of establishing rapport with consumers when engaging in suicidal conversations [35, 36]. Research among mental healthcare nurses has shown that therapeutic engagement including rapport, active listening, empathy and trust can improve the quality of care and may be associated with a reduction or resolution of suicidal crisis [37]. Pharmacists are in a unique position to recognise potential early signs of mental health crisis and/or worsening mental illness. Given the regular contact between pharmacists and consumers, pharmacy staff members in the study reflected that an established relationship with consumers can help create trust and thereby increase the likelihood that a consumer would disclose their suicidal thoughts. MHCEs also felt that pharmacists’ ability to listen and communicate nonjudgmentally could help establish a positive relationship between pharmacists and consumers.

This study suggests that pharmacists with appropriate training can ask specific questions and use direct terminology such as “suicide” or “killing yourself” to assess a person’s suicide risk, in line with MHFA principles [1]. However, the results from the qualitative analysis showed that confidence varied among pharmacy staff and many still felt uncomfortable conducting a mental health crisis assessment despite having completed MHFA training. This is consistent with other studies that showed that pharmacists were hesitant to directly ask about suicidal ideation after MHFA training [7], and suggests that opportunities to practice post-training are needed. For example, Luckie et al. demonstrated that the improvements seen in asthma first-aid knowledge did not translate to effective asthma first-aid skills [38]. Furthermore, participants who received the scenario-based training showed significant improvements asthma first aid skills, compared to conventional didactic training methods [39]. Similarly, Boukouvalas et al. demonstrated that pharmacy students who participated in MHFA training and either observed or participated in SPRPs maintained confidence improvements at follow-up than those who only completed MHFA training [18]. While these studies do support the importance of opportunities to practice skills post-training and the importance of scenario-based learning, future studies should explore other factors that may influence pharmacists’ confidence in conducting mental health crisis assessments. Longitudinal studies are also needed to investigate whether MHFA-trained participants’ crisis assessment skills change over time.

## Strengths and limitations

To our knowledge, this is the first study to provide pharmacists with an opportunity to demonstrate their MHFA skills and explore their experiences in providing MHFA post-training. Nonetheless, several potential limitations should be considered when interpreting the findings. First, the study was targeted towards pharmacy staff with MHFA accreditation so the results and experiences from the participants may not be generalisable to other study populations. Second, pharmacists’ performances observed during the SPRPs may not reflect the real-life practice of pharmacists when faced with mental health crisis situations. It is important to also note that there are other mental health crisis situations that were not explored in this study. Therefore, future research should include SPRPs of other mental health crises, to explore and compare pharmacists’ skills across a range of potential mental health crisis scenarios. Third, the differences in each MHCE’s confidence and skills in enacting the scenarios, particularly if the scenario did not reflect their personal lived experience may compromise internal validity. Nonetheless, the MHFA literature generally lacks research exploring observed behaviours and is heavily reliant on self-reported evaluations [17, 40], and this study helps build the evidence base in this area.

## Conclusion

This study provided MHFA-trained pharmacists with opportunities to practice conducting mental health crisis assessments through SPRPs. The involvement of MHCEs added authenticity to the learning experience and allowed pharmacists to engage in discussions with people with lived experience and reflect on their perspective. Participating pharmacy staff reflected on the importance of using appropriate terminology, establishing a positive pharmacist-consumer relationship and strong communication skills to overcome the challenges related to mental health crisis assessment. There is a need for additional research to examine if pharmacists’ performances in SPRPs translate to real-life practice changes and ultimately improved mental health outcomes.

## Data Availability

The datasets generated during and/or analysed during the current study are not publicly available as consent was not obtained for public sharing of data.
